# A theoretical modeling framework for motile and colonial harmful algae

**DOI:** 10.1002/ece3.9042

**Published:** 2022-07-03

**Authors:** Jackie Taylor, M. Carme Calderer, Miki Hondzo, Vaughan R. Voller

**Affiliations:** ^1^ St. Anthony Falls Laboratory Minneapolis Minnesota USA; ^2^ Department of Civil, Environmental and Geo Engineering University of Minnesota, Twin Cities Minneapolis Minnesota USA; ^3^ School of Mathematics University of Minnesota, Twin Cities Minneapolis Minnesota USA

**Keywords:** aggregation dynamics, cyanobacteria, harmful algal bloom, *Microcystis aeruginosa*, theoretical biology, vertical motility

## Abstract

Climate change is leading to an increase in severity, frequency, and distribution of harmful algal blooms across the globe. For many harmful algae species in eutrophic lakes, the formation of such blooms is controlled by three factors: the lake hydrodynamics, the vertical motility of the algae organisms, and the ability of the organisms to form colonies. Here, using the common cyanobacterium *Microcystis aeruginosa* as an example, we develop a model that accounts for both vertical transport and colony dynamics. At the core of this treatment is a model for aggregation. For this, we used Smoluchowski dynamics containing parameters related to Brownian motion, turbulent shear, differential setting, and cell‐to‐cell adhesion. To arrive at a complete description of bloom formation, we place the Smoluchowski treatment as a reaction term in a set of one‐dimensional advection‐diffusion equations, which account for the vertical motion of the algal cells through molecular and turbulent diffusion and self‐regulating buoyant motion. Results indicate that Smoluchowski aggregation qualitatively describes the colony dynamics of *M. aeruginosa*. Further, the model demonstrates wind‐induced mixing is the dominant aggregation process, and the rate of aggregation is inversely proportional to algal concentration. Because blooms of *Microcystis* typically consist of large colonies, both of these findings have direct consequences to harmful algal bloom formation. While the theoretical framework outlined in this manuscript was derived for *M. aeruginosa*, both motility and colony formation are common among bloom‐forming algae. As such, this coupling of vertical transport and colony dynamics is a useful step for improving forecasts of surface harmful algal blooms.

## Graphical abstract

Many harmful bloom‐forming algal species are motile and colonial. The theoretical modeling framework presented herein uses kinetic coagulation theory to couple passive transport via mixing, active transport via buoyancy regulation, and colony dynamics of a common cyanobacterium. Results‐‐which indicate colony dynamics are highly sensitive to algal concentration and wind speed and buoyancy‐driven transport is highly sensitive to colony dynamics‐‐have dramatic implications for bloom formation.

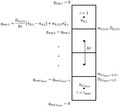

## 1 INTRODUCTION

*Microcystis aeruginosa* is a common toxin‐producing cyanobacterium capable of forming harmful algal blooms (HABs). HABs threaten both ecological and public health, and they are expected to increase in distribution, frequency, and severity as a result of climate change (O'neil et al., 2012). Predicting the timing of bloom formation has been challenging, but researchers in the field have reached consensus on general trends leading up to a HAB. A study of the record‐breaking Lake Erie algae bloom of 2011 determined that—in addition to excessive nutrient loading—quiescent meteorological conditions allowed the bloom to form and proliferate to such a massive extent (Michalak et al., 2013), a finding that has been corroborated in many subsequent studies of cyanobacteria HABs (Wells et al., 2015). Using a Bayesian biophysical model with a high‐frequency dataset, Del Giudice et al. (2021) were able to quantitatively show that quiescent conditions are not enough: High surface water temperatures and high irradiation are also necessary for bloom formation. Recently, it has been suggested that vertical heterogeneity (i.e., subsurface peaks) of *M. aeruginosa* concentration is an important precursor to *Microcystis* surface bloom formation (Seegers et al., 2015; Xiao et al., 2018; Wilkinson et al., 2019; Taylor et al., 2021). Therefore, it is reasonable to assume improving models for the drivers of *M. aeruginosa* vertical distributions will likely lead to improved predictions of HAB timing.

There are two key traits related to the ubiquity of *M. aeruginosa*: vertical motility and colony formation. Vertical motility is achieved through algal cell buoyancy regulation via intracellular gas vesicles. Under low levels of mixing, *M. aeruginosa* sinks to lower light intensities during the day and floats towards the water surface at night, although a critical water temperature threshold must be reached in order for cells to regain buoyancy (Ibelings et al., 1991; Thomas & Walsby, 1985, 1986). Once that threshold is reached, increasing temperature increases buoyant velocity (You et al., 2018). Vertical motility gives *M. aeruginosa* a particular advantage in stratified lake environments. Stratified lakes are characterized by three distinct layers: The epilimnion or surface mixed layer is the hot, well‐mixed surface layer; the hypolimnion is the cold, well‐mixed bottom layer; and the metalimnion is the intermediate layer of steep temperature gradient connecting the epilimnion to the hypolimnion. Using the three‐dimensional ecological‐hydrodynamic modeling software ELCOM‐CAEDYM, Chung et al. (2014) were able to demonstrate a shallow mixed layer depth (close to the photic depth) favored buoyant cyanobacteria dominance, indicating lake thermal structure controls algal population dynamics.

Colony dynamics remain rather illusive, but colonies have been demonstrated to form in the presence of grazers, low to medium turbulence, and low nutrient conditions. Colonies formed by reproduction and growth tend to be compact, whereas colonies that form by collisions tend to be fractal. There is also a well‐documented progression from a unicellular morphology in the spring to a fractal colonial morphology in the summer (Xiao et al., 2018). In a field study, Cao and Yang (2010) found that large colonies (greater than 20 cells per colony) did not appear until May but composed 90% of cells in a June surface bloom. They also calculated the mean number of cells in the surface bloom to be about 120 cells/colony. Between field work and experiments, Qin et al. (2018) found that wind promotes aggregation, creating heterogeneous size distributions in *Microcystis* populations.

There are two threads of previous models to follow. There are models that describe aggregation processes of phytoplankton, and there are models that describe the vertical motility of *M. aeruginosa*. To describe the aggregation processes of phytoplankton, models use Smoluchowski aggregation terms (Ackleh & Miller, 2018; Jackson, 1990; Smoluchowski, 1917). Because these models typically have applications in wastewater treatment or marine snow, the only transport considered is the loss of aggregates via sinking out of the surface mixed layer (Engel et al., 2004; Lee et al., 2000; Teh et al., 2016).

Early models of *Microcystis* motility use light intensity as a driver of changes in individual cell density—high light intensities lead to an increase in cell density, whereas low light intensities lead to a decrease in cell density. The buoyant velocity of cells is then calculated through a modified Stokes settling velocity that is governed by the difference between algal cell density and the surrounding water density (Wallace et al., 2000). Turbulent transport has since been incorporated into these models (Medrano et al., 2013; Zhu et al., 2018). By combining their model with principal component analysis, Feng et al. (2018) demonstrated that turbulence‐induced mixing explained over half of the variability of early surface bloom formation, and that buoyancy regulation was more important for bloom maintenance and formation of late‐season blooms. Although the transport of different (fixed) colony sizes is investigated in the aforementioned *Microcystis* motility models, they do not incorporate aggregation dynamics, despite the well‐documented progression from unicellular to colonial morphologies.

In a previous field study, statistical methods were used to elucidate the reliance of *Microcystis*‐dominated algal vertical distributions on lake thermal stratification variables (Taylor et al., 2021). Following the protocol discussed in Vinatier et al. (2011), which suggests using statistical and mechanistic models in an iterative manner to uncover forcings of spatial heterogeneity, we propose a mechanistic model to analyze the effects of hydrodynamic and biological processes underlying the spatial patterns observed in the previous field study. The primary objective of this model is not to replicate exact field observations but to instead generate hypotheses for the biophysical drivers of general field trends and observations. To this end, we couple algal cell aggregation dynamics with algal motility in a system of one‐dimensional partial differential equations that capture lake hydrodynamics to investigate the role of the colony and motility dynamics on *M. aeruginosa* surface bloom formation.

## 2 METHODS

### 2.1 Aggregation preliminaries

**FIGURE 1:**
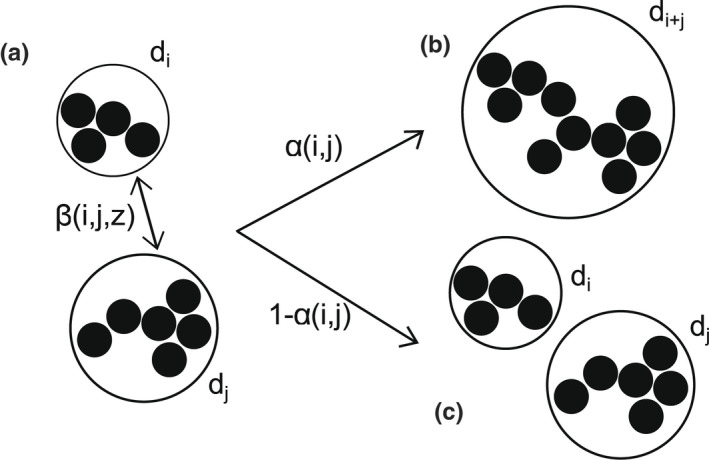
Schematic of aggregation. Circles indicate the equivalent spherical diameter, *d*_*i*_, of the fractal aggregate of size *i*. (a) Two aggregates of size *i* and *j* collide. This collision can either result in (b) aggregation and the formation of a *i* + *j* sized aggregate, or (c) collision without aggregation. Rate of collisions is controlled by *β*(*i*,*j*,*z*), but the number of collisions that result in aggregation is controlled by *α*(*i*,*j*)

In the absence of any advective or diffusive transport, discrete aggregation dynamics can be described by the Smoluchowski coagulation model (Smoluchowski, 1917):

(1) $$\frac{dn_{k}}{dt}=\frac{1}{2}\sum_{i+j=k}\alpha \left(i,j\right)\beta \left(i,j\right)n_{i}n_{j}-\sum_{i=1}^{\infty }\alpha \left(i,k\right)\beta \left(i,k\right)n_{i}n_{k}$$

where *n*_*k*_(*z*,*t*) is the concentration of an aggregate of size *k*, *α*(*i*,*j*) is the sticking probability and *β*(*i*,*j*) is referred to as the aggregation, or coagulation, kernel of particles of size *i* and *j* (Figure 1). Occasionally the product of *α*(*i*,*j*) and *β*(*i*,*j*) is referred to as the aggregation kernel, instead of just *β*(*i*,*j*). We leave the two parameters decoupled mainly for the sake of visualizing the process (Figure 1) but also to conceptually differentiate the hydrodynamic drivers of *β*(*i*,*j*) (Equations (2), (3), (4), (5)) from the biological drivers of *α*(*i*,*j*) (Section 2.2.2). The first term on the right‐hand side describes the formation of a *k*‐sized aggregation, whereas the second term on the right‐hand side describes the loss of a *k*‐sized aggregation through the formation of a *k* + *i*‐sized aggregate. An infinitely‐sized particle represents a loss of mass due to gelation. Equation 1 has had far‐reaching applications in addition to phytoplankton modeling, from aerosols to random graph theory and polymerization to planet formation (Aldous, 1999).

While analytical solutions exist for some simple aggregation kernels ($\beta \left(i,j\right)\sim 1,\;\beta \left(i,j\right)\sim i+j$, and $\beta \left(i,j\right)\sim \mathrm{ij}$), realistic aggregation kernels are rarely analytically tractable. In the present context, *β*(*i*,*j*) is calculated as the sum of aggregation kernels for Brownian motion, *β*_Br_(*i*,*j*,*z*), turbulent shear, *β*_TS_(*i*,*j*,*z*), and differential settling, *β*_DS_(*i*,*j*,*z*), each, respectively, defined as (Ackleh & Miller, 2018; Thomas et al., 1999)

(2) $$\beta_{\mathrm{Br}}\left(i,j,z\right)=\frac{2T\left(z\right)k_{B}{\left(d_{i}+d_{j}\right)}^{2}}{3\mu \left(z\right)\left(d_{i}d_{j}\right)}$$

(3) $$\beta_{\mathrm{TS}}\left(i,j,z\right)=\frac{4G\left(z\right){\left(d_{i}+d_{j}\right)}^{3}}{3}$$

and

(4) $$\beta_{\mathrm{DS}}\left(i,j,z\right)=\pi {\left(d_{i}+d_{j}\right)}^{2}\left|w_{i}\left(z\right)-w_{j}\left(z\right)\right|$$

such that

(5) $$\beta \left(i,j,z\right)=\beta_{\mathrm{Br}}\left(i,j,z\right)+\beta_{\mathrm{TS}}\left(i,j,z\right)+\beta_{\mathrm{DS}}\left(i,j,z\right)$$

where *T*(*z*) is the water temperature (K), *k*_B_ is Boltzmann's constant (1.38 × 10^−23^ m^2^ kg s^−2^ K^−1^), *μ*(*z*) is the dynamic viscosity of water (kg/m/s), $G\left(z\right)={\left(\frac{\epsilon }{\nu }\right)}^{\frac{1}{2}}$ is the turbulent shear rate (1/s), *ϵ*(*z*) is the rate of turbulent kinetic energy dissipation (m^2^/s^3^), and $\nu \left(z\right)$ is the kinematic viscosity of water (m^2^/s). The equivalent spherical diameter of a colony of size *i*, *d*_*i*_ (m), is given by

(6) $$d_{i}=\frac{i^{\frac{1}{D_{f}}}d_{0}}{\phi }$$

where *D*_*f*_ = 2.5 is the fractal dimension (Nakamura et al., 1993), *d*_0_ = 5 μm is the diameter of a single cell of *M. aeruginosa* (Xiao et al., 2018), and *ϕ* is the colony porosity that linearly decreases from *ϕ* = 1 for single cells and *ϕ* = 0.2 for colonies of size *k*_max_ (Medrano et al., 2013). Equation (2) is derived from thermodynamic principles of Brownian motion, Equation (3) defines the rate of collisions for sub‐Kolmogorov particles in turbulent flow (i.e., the largest aggregate diameter is smaller than the length scale of the smallest turbulent eddies), and Equation (4) describes collisions as a result of different‐sized aggregates moving at different velocities. Aggregation due to Brownian motion is typically much slower than aggregation due to turbulent shear, and aggregation due to differential settling will be large for aggregates of drastically different sizes but will be small for aggregates of close to the same size.

There are several assumptions of this formulation that should be addressed before continuing.

1. We assume diffusion‐limited aggregation rather than reaction‐limited aggregation, meaning the aggregation process will be limited by diffusion due to Brownian motion and not by the sticking probability of collisions. This is reasonable for colony‐forming species of algae in a system where the domain size is much larger than the aggregate sizes.
2. We assume a maximum colony size, below which there will be no disaggregation—colonies cannot split up once formed. Effectively, we assume any colonies above the maximum colony size instantaneously disaggregate into their constituent parts. These assumptions are validated by the lab experiments of O'Brien et al. (2004), which demonstrated disaggregation of *M. aeruginosa* is negligible for the size range of aggregates being modeled subjected to expected field turbulence conditions.
3. We assume aggregates grow in size through particle collisions only. When aggregates consist of living organisms, it is possible for aggregates to increase in size through cell growth and reproduction in addition to particle collisions. However, it is hypothesized that the fractal colonies of *M. aeruginosa* are formed primarily through collisions, so we neglect aggregation due to cell growth (Xiao et al., 2018).
4. We assume aggregation is uniform over any given horizontal cross‐section in order to facilitate the construction of a one‐dimensional model.

### 2.2 The mathematical model

To provide a biophysical mechanistic understanding of field vertical distributions of colonial and motile harmful algae, we develop the following model to couple colony formation with the vertical transport of *M. aeruginosa*. Let *n*_*k*_(*z*,*t*) be the number of colonies containing *k* cells of *M. aeruginosa* per unit volume (colonies/m^3^), *t* be time (s), *z* be depth (m), *D*_*Z*_(*z*) be the sum of molecular diffusion and turbulent dispersion coefficients (m^2^/s), *w*_*k*_(*z*,*t*) be the buoyant velocity of a colony containing *k* cells of *M. aeruginosa* (m/s), *β*(*i*,*j*,*z*) be the Smoluchowski aggregation kernel for colonies of size *i* and *j* at a depth *z* defined by Equation (5) (m^3^/s), and *k*_max_ be the maximum number of cells in a single colony. If we assume nutrients are not limiting, then we suggest that the combined vertical transport and aggregation of a colony of size *k* can be described by the following advection‐dispersion‐reaction equation:

(7) $$\frac{\partial n_{k}}{\partial t}=\frac{\partial }{\partial z}\left(D_{Z}\frac{\partial n_{k}}{\partial z}\right)-\frac{\partial }{\partial z}\left(w_{k}n_{k}\right)+\frac{1}{2}\sum_{i+j=k}\alpha \left(i,j\right)\beta \left(i,j,z\right)n_{i}n_{j}-\sum_{i=1}^{k_{\max}-k}\alpha \left(i,k\right)\beta \left(i,k,z\right)n_{i}n_{k}$$

with boundary conditions

(8) $$\begin{array}{c} \frac{\partial n_{k}}{\partial z}{{|}_{z=0}=\frac{\partial n_{k}}{\partial z}|}_{z=h_{\max}}=0 \end{array}$$

and piecewise uniform initial conditions given by

(9) $$n_{k}\left(z,0\right)=n_{k}^{0}\left(z\right)=\left\{\begin{array}{ll} 2.3\times 10^{7}\;\text{colonies}/m^{3} & k=1\\ 0 & k>1\\ 0 & z>h_{\mathrm{ML}}\;\forall k \end{array}\right.$$

where *z* = 0 at the air‐water interface, *z* = *h*_max_ at the lakebed, and *h*_ML_ is the width of the surface mixed layer. The no‐flux boundary conditions ensure cells cannot leave the water column through atmospheric or soil exchange. Due to the seasonal progression of *M. aeruginosa* from unicellular to colonial morphology, we begin simulations with only single cells. Since we are typically more interested in overall *M. aeruginosa* concentration profiles rather than the concentration profiles of any given colony size, we convert concentrations of colonies of size *k* to total *M. aeruginosa* concentration by

(10) $$C\left(z,t\right)=\sum_{k}{\mathrm{kn}}_{k}\left(z,t\right)$$

where *C*(*z*,*t*) is the total concentration of *M. aeruginosa* (cells/m^3^). Note that we have a discrete number of total cells in the system, but both concentration and time are continuous. Using the aforementioned relationships for the aggregation kernel, appropriate forms for the sticking probability and diffusion coefficient, and the specification of an expression for the settling velocity, *w*_*k*_(*z*,*t*), we can readily develop a numerical simulation of Equation (7).

#### 2.2.1 System details

For *M. aeruginosa*, the largest stable colony size varies between 220–420 μm, depending on the rate of turbulent kinetic energy dissipation in the water column (O'Brien et al., 2004). Meaning for colonies of diameters smaller than 220 μm, we assume fragmentation is negligible for all reasonable environmental conditions. Using the aggregation parameters listed in Section 2.1, this diameter roughly corresponds to a colony of size *k* = 580 cells/colony. To explore the features of the model in a numerically efficient manner, we have cut off the colony size domain at *k*_max_ = 101 cells/colony, which corresponds to a maximum colony diameter of *d*_101_ = 160 μm. This is approximately half the average maximum colony diameter determined by (O'Brien et al., 2004), and the mean colony size that Cao and Yang (2010) measured in a *Microcystis* HAB. Further, diameters larger than this size may exceed the Kolmogorov length scale, thereby compromising the validity of Stokes' law and leading to the overestimation of buoyant velocities (Medrano et al., 2013).

**FIGURE 2:**
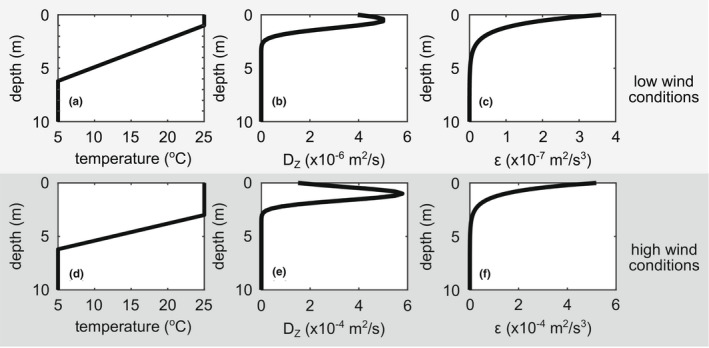
Smoothed field data. Low wind profiles for (a) temperature, (b) turbulent dispersion coefficient, *D*_*Z*_, and (c) rate of turbulent kinetic energy dissipation, *ϵ*. high wind profiles for (d) temperature, (e) turbulent dispersion coefficient, *D*_*Z*_, and (f) rate of turbulent kinetic energy dissipation, *ϵ*. Note the differences in orders of magnitude for *D*_*Z*_ and *ϵ* under low wind and high wind conditions. Low wind conditions roughly correspond to wind speeds of 2 m/s, whereas high wind conditions roughly correspond to wind speeds of 8 m/s

Recall *M. aeruginosa* typically thrives in stratified lake environments. As such, the model must incorporate depth‐dependent water temperature, water density, and turbulence profiles. To get a sense of how the model behaves in field conditions, we used data collected by a Self‐Contained Autonomous MicroProfiler (SCAMP) from Ramsey Lake. Ramsey Lake (45.2073°N, 93.9969°W) is a stratified and eutrophic lake in Minnesota, USA with a maximum depth of approximately 24 m, a surface area of approximately 1.3 km^2^, and a history of *M. aeruginosa* blooms (Rao & Hsu, 2008). SCAMP records temperature fluctuations throughout the water column. Following the protocol in Chen et al. (2001), estimated spectra were calculated using Batchelor curve fitting, which were then used to calculate turbulent kinetic energy dissipation rates. From this dataset, profiles for water temperature, *D*_*Z*_, and *ϵ* were constructed from field data under low wind conditions and high wind conditions (Figure 2). The low wind data were obtained on August 2nd, 2018 11:22:20—the maximum value of *ϵ* was measured to be 3 × 10^−7^ m^2^/s^3^ during surface wind speeds of approximately 2.3 m/s. The high wind data were obtained on August 30th, 2018 11:34:26—the maximum value of *ϵ* measured was 4 × 10^−4^ m^2^/s^3^ and corresponded to wind speeds of approximately 8 m/s. To put these choices in context, typical values of *ϵ*(*z*) in the field range from 10^−11^ to 10^−6^ m^2^/s^3^, and typical values of *D*_*Z*_(*z*) range from 10^−6^ to 10^−2^ m^2^/s (Wüest & Lorke, 2003).

Since *M. aeruginosa* buoyancy is largely mediated by light intensity, we must also construct diurnal light profiles. We generated surface light intensities, *I*_0_(*t*), by

(11) $$I_{0}\left(t\right)=I_{\max}\sin\frac{\mathrm{\pi t}}{D_{L}}$$

where *I*_max_ is the maximum surface light intensity and *D*_L_ is the photoperiod. To best replicate previous models, values of *I*_max_ = 800 W/m^2^ and *D*_L_ = 16 h were chosen (Medrano et al., 2013). Depth‐dependent light intensities, *I*(*z*,*t*), can then be calculated by

(12) $$I\left(z,t\right)=I_{0}\left(t\right)e^{-k_{I}z}$$

where *k*_I_ is the light attenuation coefficient (*k*_I_ = 1.3 m^−1^ (Medrano et al., 2013)).

#### 2.2.2 Biological parameters

**FIGURE 3:**
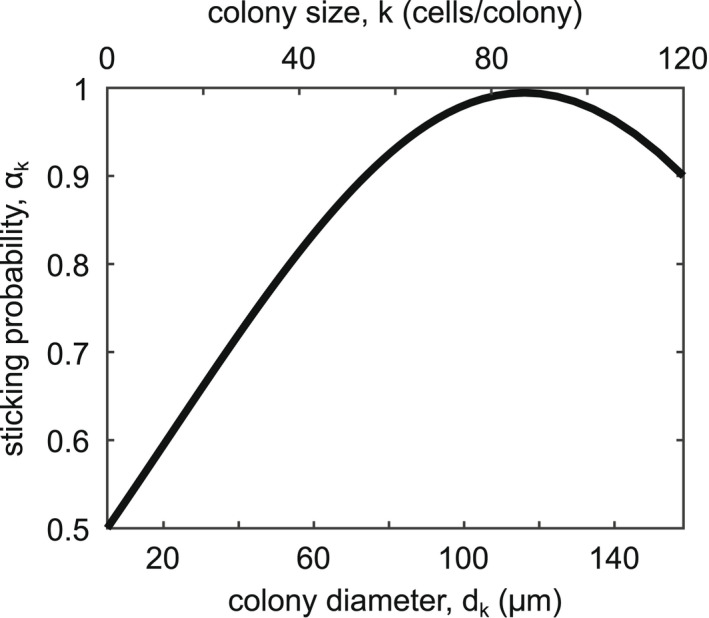
New figure to elaborate on the sticking probability function. Sticking probability, *α*_*k*_, vs colony diameter, *d*_*k*_ (μm), and colony size, *k* (cells/colony), where *α*_*k*_ is defined by $\alpha_{k}\left(d_{k}\right)=0.994e^{-{\left(\frac{\left(d_{k}-0.000116\right)}{0.000134}\right)}^{2}}$. Single cells will aggregate upon colliding 50% of the time, whereas colonies of size *k* = 95 cells/colony will always aggregate after collisions. Note that $\alpha \left(i,j\right)=\max\left\{\alpha_{i},\alpha_{j}\right\}$

Let us address the sticking probability, *α*(*i*,*j*). Previous models of Smoluchowski aggregation have related *α*(*i*,*j*) to the fractal dimension of aggregates or to the estimated number of particles near the aggregate (Schmitt et al., 2000; Zidar et al., 2018). This particular situation warrants a more biological approach. *M. aeruginosa* uses extracellular polysaccharides (EPS) as adhesive during the aggregation process; therefore, it is reasonable to assume sticking probability will increase with EPS content. Zhu et al. (2014) determined that, in field samples of *M. aeruginosa*, EPS content peaks at colony diameters between 100 and 150 μm. Interestingly, these diameters are similar to the average colony size found in *Microcystis* HABs (Cao & Yang, 2010). Using this, we define a function that gives the sticking probability of a colony of size *k*, *α*_*k*_ = *f*(*d*_*k*_), which achieves a minimum value of *α*_*k*_ = 0.5 at *d*_1_ = 5 μm and a maximum value of *α*_*k*_ = 1 at *d*_95_ = 125 μm (Figure 3). To calculate the sticking probability for a collision between a colony of size *i* and size *j*, we define $\alpha \left(i,j\right)=\max\left\{\alpha_{i},\alpha_{j}\right\}$. Larger colonies will therefore be ‘stickier’ than small colonies, so more of their collisions will result in aggregation.

The buoyant velocity, *w*_*k*_, is calculated using subroutines described in previous models, which (i) relate light intensity to individual cell density, then (ii) relate individual cell density to colony density using the fractal dimension of *M. aeruginosa* aggregates, then (iii) use the colony density to calculate a modified Stoke's velocity (Medrano et al., 2013; Nakamura et al., 1993; Wallace et al., 2000) by

(13) $$w_{k}=\frac{{\mathrm{gd}}_{k}^{2}\left(\frac{\rho_{k}}{\rho_{W}}-1\right)}{18\nu }$$

where *ρ*_*k*_ is the density of a colony of size *k*. Subroutine details to calculate *ρ*_*k*_ can be found in Appendix A. We use the same equations and parameter values used in the work of Medrano et al. (2013), with a modification for the ratio of cell volume to colony volume that accounts for the fractal geometry of aggregates and the relationship between EPS content and colony size. We expect sinking during the day (positive *w*_*k*_) and floating at night (negative *w*_*k*_), although velocity magnitudes and general transport dynamics will vary across colony size. In experiments, You et al. (2018) recorded buoyant velocities of 10^−6^ m/s at 17.5*°*C and 10^−5^ m/s at 28*°*C for small colonies. For large colonies, buoyant velocities have been recorded as large as 10^−3^ m/s (Wallace et al., 2000).

#### 2.2.3 Numerical considerations

**FIGURE 4:**
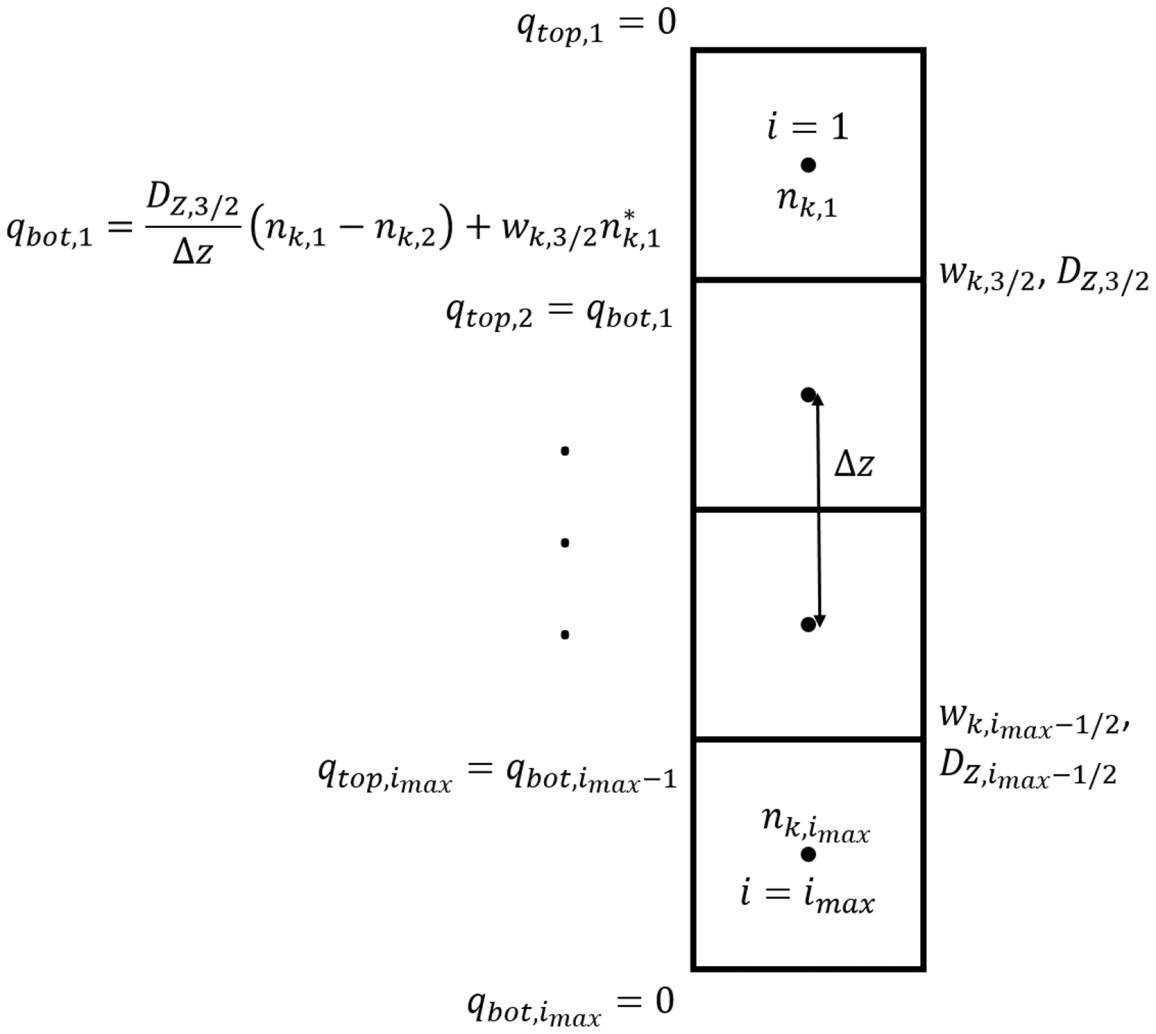
Schematic of numerical scheme. Fluxes, *q*_top_,_*i*_ and *q*_bot_,_*i*_, are calculated as the sum of diffusive and advective fluxes at grid cell interfaces, but concentrations are calculated at grid cell node points. $n_{k,i}^{*}$ depends on the sign of *w*_*k*_,_*i*_ and is defined in Equation (15). Since we can calculate the new concentration of a colony of size *k* in grid cell *i* at time step *m* by 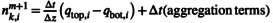, conservation of mass is ensured by setting *q*_top_,_*i* + 1_ = *q*_bot_,_*i*_. To satisfy boundary conditions, fluxes at the top of the first grid cell and at the bottom of the last grid cell are defined to be zero for all time

We are using an explicit forward‐in‐time upwind numerical scheme with fluxes defined at grid cell interfaces and concentrations defined at grid cell node points (Figure 4). For a given grid cell *i* at time step *m*, the new concentration of colonies of size *k* in that grid cell is calculated as

(14) $$\begin{array}{l} n_{k,i}^{m+1}=n_{k,i}^{m}+\frac{\Delta t}{\Delta z}\left(\frac{D_{i-\frac{1}{2}}}{\Delta z}\left(n_{k,i-1}^{m}-n_{k,i}^{m}\right)+w_{k,i-\frac{1}{2}}^{m}n_{k,i}^{*m}\right)\ldots \\ \ldots -\frac{\Delta t}{\Delta z}\left(\frac{D_{i+\frac{1}{2}}}{\Delta z}\left(n_{k,i}^{m}-n_{k,i+1}^{m}\right)+w_{k,i+\frac{1}{2}}^{m}n_{k,i+1}^{*m}\right)+\Delta t\left(\text{aggregation terms}\right) \end{array}$$

where the subscripts $i\pm \frac{1}{2}$ denote parameters defined at the top or bottom interface of grid cell *i*, the aggregation terms are defined by Equations ((2), (3), (4), (5)), and

(15) $$n_{k,i}^{*m}=\left\{\begin{array}{ll} n_{k,i-1}^{m} & w_{k,i}^{m}\geq 0\\ n_{k,i}^{m} & w_{k,i}^{m}<0 \end{array}\right.$$

by upwinding.

Table 1 shows numerical parameter values used for all simulations. The time step, Δ*t*, was chosen to be small enough to ensure the stability of the numerical scheme, and the grid cell width, Δ*z*, was chosen to be small enough to minimize numerical dispersion of the upwind scheme while also maintaining stability. To address numerical dispersion, we tested the time to large colony appearance for the parameters described in Table 1 against a finer grid size. In the base case simulation, large colonies appear in 13.4 days; if we instead use Δ*z* = 0.1 m (and a correspondingly smaller time step of Δ*t* = 5 s), large colonies appear in 16.1 days. This three‐day slowdown indicates that our scheme is not completely devoid of numerical dispersion. However, the goal of this manuscript is first and foremost to investigate the applicability of Smoluchowski aggregation to describe *M. aeruginosa* colony dynamics—not to solve the inverse problem of parameter estimation or make predictions with a real dataset. In this sense, we feel that our choices of space and time steps efficiently capture the correct physical behaviors and provide an appropriate order of magnitude prediction for the timing and appearance of large colony sizes.

**TABLE 1:** Numerical parameters

Variable	Description	Value
Δ*z*	Grid cell width	0.2 m
Δ*t*	Time step	10 s
*z*_max_	Maximum depth of domain	10 m

## 3 RESULTS

### 3.1 Appearance and distribution of colonies

We will start with the simplest simulation that still allows for the investigation of important model features: six weeks of a repeating photoperiod and constant lake thermal and hydrodynamic profiles (Table 2). The repeating photoperiod is generated by Equations (11) and (12); the constant lake thermal and hydrodynamic profiles are shown in Figure 2d–f. For the base case simulation, the lake thermal and hydrodynamic profiles represent high wind conditions. Field data indicate *Microcystis* can transition from a predominantly unicellular morphology to a predominantly colonial morphology over a monthly period (Cao & Yang, 2010; Xiao et al., 2018), so a six‐week simulation time was chosen to ensure aggregation would be evident. Using the conditions outlined in Table 2, Equation (13) predicted buoyant velocities ranging from −10^−4^ (floating) to 10^−3^ m/s (sinking) and Equations ((2), (3), (4), (5)) predicted aggregation kernels in the range *β*(*i*,*j*,*z*) ∈ [10^−13^, 10^−9^] m^3^/s.

**TABLE 2:** Base case simulation conditions

Condition	Description	Further details
Motility	Regulated by light‐dependent buoyancy	Equation (13)
Meteorological forcings	Constant high wind and lake thermal profile	Figure 2d–f
Sticking probability	*α*(*i*,*j*) ∈ [0.5,1] with peak at *d*_95_ = 125 μm	Section 2.2.2
Initial algal concentration	Only single cells in mixed layer	Equation (9)

**FIGURE 5:**
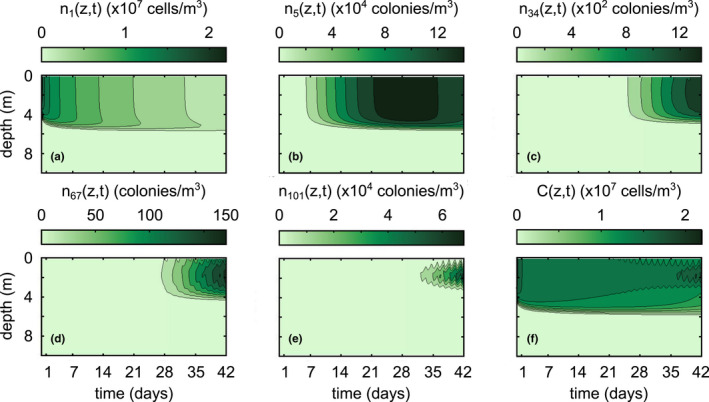
Concentration profiles over six weeks of simulation during high wind conditions (shown in Figure 2d–f) for (a) *n*_1_(*z*,*t*), (b) *n*_5_(*z*,*t*), (c) *n*_34_(*z*,*t*), (d) *n*_67_(*z*,*t*), (e) *n*_101_(*z*,*t*), and (f) *C*(*z*,*t*). Color bar changes scale for each subfigure. The wiggles visible in (d)–(e) show the diurnal migration of large‐sized colonies

The model demonstrates small colonies will diffuse throughout the mixed layer (Figure 5a–c), but large colonies exhibit diurnal migrations to a depth with preferred low light intensity (Figure 5d,e). In general, small colonies will lose mass as they aggregate into larger colonies, which gain mass. Medium‐sized colonies never achieve high mass (Figure 5c,d), and colonies of size *k* = 101 appear before colonies of size *k* = 67. This indicates large colonies aggregate with each other faster than they aggregate with small colonies, a finding consistent with coagulation kinetic theory (Smit et al., 1994). The overall concentration profile, *C*(*z*,*t*) (Equation 10), is mostly influenced by large colonies by approximately the fifth week of simulation (Figure 5f).

### 3.2 Factors affecting vertical distribution

**FIGURE 6:**
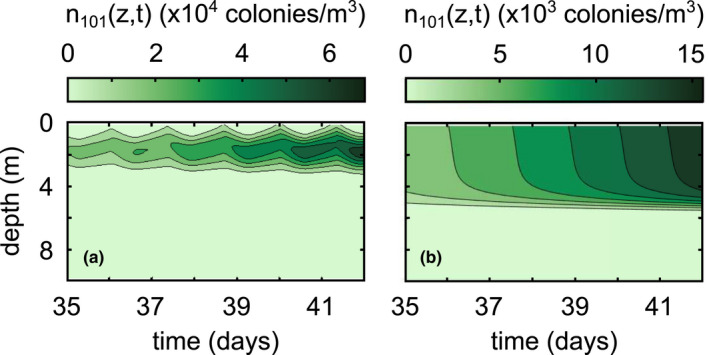
Differences in vertical distributions of large‐sized colonies between (a) the base case simulation in Figure 5 and (b) turning off advection by setting *w*_*k*_(*z*,*t*) ≡ 0

While advection is negligible for single cells and small colonies, motility plays a key role in the vertical distribution of large‐sized colonies (Figure 6). The time it takes for large colonies to appear is approximately equivalent to whether advection is on or off, but the inclusion of motility allows the large colonies to migrate to a preferred depth of low light intensity (Figure 6a).

**FIGURE 7:**
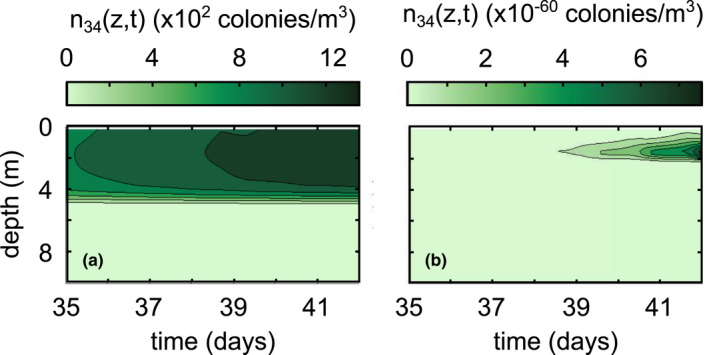
Differences in vertical distributions of colonies of size *k* = 34 between (a) the base case simulation in Figure 5 and (b) low wind conditions (Figure 2)

We also see changes in vertical distributions when we change wind conditions (Figure 7). During high wind conditions, small colonies become uniformly distributed throughout the mixed layer. If we instead run the simulation under constant low wind conditions shown in Figure 2a–c, smaller colonies (e.g., *k* = 34) are able to advect to a preferred depth of low light intensity, although their diurnal migrations are not as pronounced (compare Figure 7b to Figure 6a or Figure 5e). In addition, wind also seems to significantly control the time it takes for colonies to appear. Synthesizing these results, high wind conditions lead to more medium‐sized colonies, but they will be well‐mixed throughout the surface mixed layer. On the other hand, low wind conditions lead to far fewer medium‐sized colonies, but the colonies will be able to concentrate around a depth of preferred low light intensity.

### 3.3 Factors affecting aggregation

**FIGURE 8:**
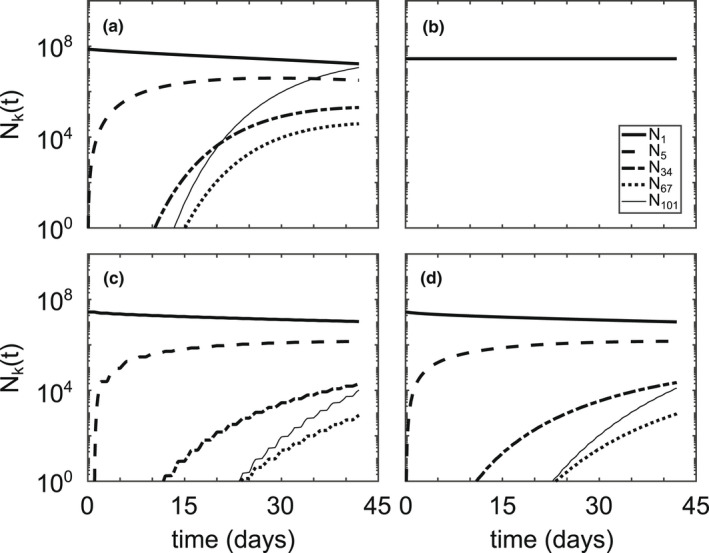
Cell count, *N*_*k*_(*t*), of various colony sizes for (a) the base case simulation (Table 2), (b) low wind conditions (Figure 2), (c) switching between high wind and low wind conditions every day, and (d) switching between high wind and low wind conditions every hour. Total number of cells is conserved for all simulations. Cell counts, *N*_*k*_, were calculated by $N_{k}=\sum_{z}kn_{k}\Delta z$

**FIGURE 9:**
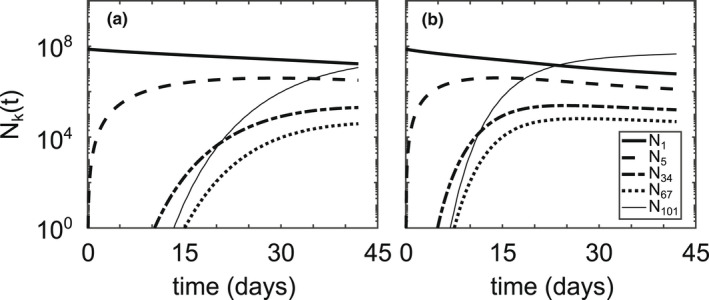
Cell count, *N*_*k*_(*t*), of various colony sizes for (a) the base case simulation from Figure 5 and (b) enforcing all collisions result in aggregation by setting *α*(*i*,*j*) ≡ 1. Total number of cells is conserved for all simulations

There are few situations less likely to occur than 6 weeks of the exact same meteorological conditions on repeat, so we must explore how the model behaves under different conditions. To this end, let us define

$$N_{k}\left(t\right)=\sum_{z}{\mathrm{kn}}_{k}\left(z,t\right)\Delta z$$

to be the total number of cells in a colony of size *k*. Since *n*_*k*_ is a continuous variable and *n*_*k*_Δ*z* is not necessarily greater than one, it is possible for *N*_*k*_ < *k*. We are more concerned when colonies of various sizes appear at some comparative concentration value rather than the actual concentration, so *N*_*k*_(*t*) acts as a suitable marker for the appearance of colonies. We can now rerun the simulation described in the previous Section 3.1 while changing one condition at a time to see how each individual change affects *N*_*k*_(*t*) for various colony sizes (Figures 8 and 9). Using low wind conditions (Figure 2) dramatically reduces aggregation—in the entire six‐week simulation, the largest colony size achieved is *k* = 3 cells/colony (Figure 8b). If we introduce transient hydrodynamic profiles that represent stepwise intermittent wind conditions between high wind 50% of the time and low wind the other 50% of the time, either on a daily or hourly time scale, aggregation is slowed down by a factor of approximately two (Figure 8c,d). Setting the sticking probability, *α*(*i*,*j*), to be unity for all colony sizes allows the large‐sized colonies to show up approximately 5 days before their appearance in the base case simulation, eventually becoming more abundant than the single cell population (Figure 9b).

**FIGURE 10:**
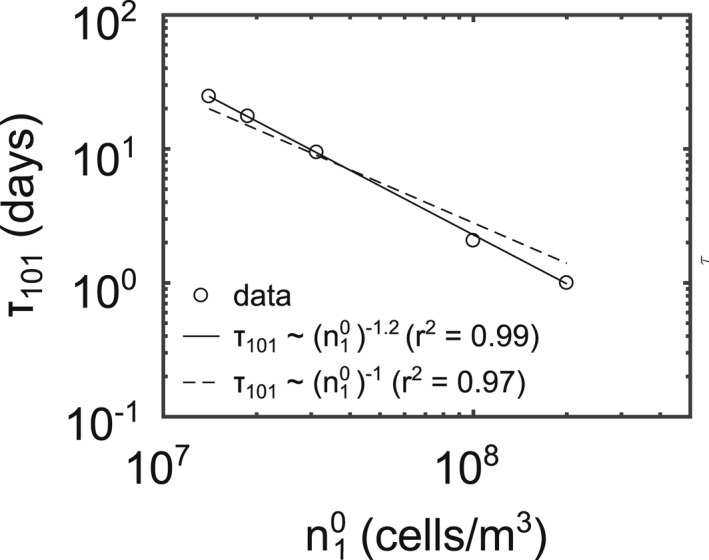
Initial concentration of singles cells within the mixed layer vs time to appearance of colonies of size *k* = 101. Both *x*‐ and *y*‐axes are log scales. Solid line shows the best fit, with a slope of −1.2 ($\tau_{101}=1.1\times 10^{10}{\left(n_{1}^{0}\right)}^{-1.2}$). Dashed lines show an exactly inversely proportional relationship between *τ* and $n_{1}^{0}$ ($\tau_{101}=2.7\times 10^{8}{\left(n_{1}^{0}\right)}^{-1}$). With a starting concentration of 1 × 10^7^ cells/m^3^, colonies of size *k* = 101 never appear within the 42‐day simulation period

Along with wind conditions, the speed of aggregation is highly sensitive to initial algal concentrations (Figure 10). Let us define *τ*_*k*_ to be the time such that *N*_*k*_(*τ*_*k*_) = 1. As long as initial algal concentrations are greater than 1 × 10^7^ cells/m^3^, then *τ*_*k*_ is approximately inversely proportional to initial concentrations within the mixed layer, $n_{1}^{0}$.

### 3.4 Summary of main results

1. For constant high wind conditions and initial uniform single cell concentrations of 10^7^ cells/m^3^ within the surface mixed layer, the largest colonies of size *k* = 101 appear in approximately 2 weeks and dominate in approximately 5 weeks.
2. Large colonies exhibit diurnal migrations, with concentration peaks located around a depth of preferred low light intensity; small colonies are dispersed throughout the surface mixed layer. The minimum colony size capable of diurnal migrations increases with increasing wind speed.
3. Aggregation is negligible during low wind conditions.
4. Intermittent wind conditions, which oscillate between high and low winds at some given frequency such that high wind conditions are achieved 50% of the time, slow the appearance of large colony sizes by a factor of two.
5. Above an initial algal concentration of 10^7^ cells/m^3^, there is a power‐law dependence between the time to appearance of large colonies and initial algal concentration.

## 4 DISCUSSION AND CONCLUSION

### 4.1 Discussion of model results

Our results generally coincide with those of existing literature, with a few important caveats. Ackleh and Miller (2018) found aggregation rates on the order of 10^−12^ m^3^/s using Smoluchowski aggregation to model phytoplankton dynamics, which is in line with those calculated in our simulations (*β*(*i*,*j*,*z*) ∈ [10^−13^,10^−9^] m^3^/s). Medrano et al. (2013) found maximum buoyant velocity magnitudes on the order of 10^−3^ m/s for large colonies, which is an equivalent order of magnitude of our maximum calculated buoyant velocities ($w_{k}\left(z,t\right)\in \left[-10^{-4},10^{-3}\right]$ m/s). Our model also predicts aggregation at a time scale that roughly corresponds with the field study by Cao and Yang (2010), wherein the dominant morphology of *Microcystis* transitioned from single cells to large colonies in about a month. The model of Medrano et al. (2013) showed that small colonies of *M. aeruginosa* are not able to overcome turbulent mixing, whereas large colonies exhibit notable daily migrations controlled by the photic depth. This is directly compatible with our model results, keeping in mind that the intensity of wind controls the minimum colony size capable of diurnal migrations (Figures 5, 6, 7). If we define the sticking probability to be unity for all colony sizes, the large‐sized colonies appear within a couple of days, much faster than they appear in field conditions (Figure 9). Relating the sticking probability to the extracellular polysaccharide content, which is in turn related to colony size, slows down aggregation to a rate consistent with field observations. These findings support the claim that Smoluchowski coagulation kinetics qualitatively describe the aggregation processes of *M. aeruginosa*.

The model unveils two important dependencies of aggregation on wind speed and algal concentration. Colony size distributions are highly sensitive to wind‐induced mixing (Figure 8), a phenomenon that was previously revealed in experiments and field work (Qin et al., 2018). Colonies of size *k* = 101 cells/colony appeared within 15 days during high wind conditions, but the largest colony size to appear during low wind conditions was *k* = 3 cells/colony (Figure 8a,b). Cutting the large wind events in half—either daily or hourly—slowed the appearance of the large‐sized colonies by a factor of two (Figure 8c,d). This implies that the speed of aggregation is directly proportional to the duration of large wind events, causing relatively short‐lived wind events to lead to rapid aggregation (recall the dependence of *β*(*i*,*j*,*z*) on the turbulent shear rate in Equation (3)).

When our model indicates aggregation is negligible for low wind conditions, it does not mean aggregation is not occurring. Instead, this indicates that processes like light‐driven motility are considerably more significant than aggregation during low wind conditions. While this may seem to disagree with the conclusions of Qin et al. (2018), which stated that low to medium turbulence is necessary to promote colony formation, this finding is actually just placing their experimental results in the context of a deep, dimictic lake. Small to moderate amounts of turbulence will in fact promote aggregation, but it does not do so at a rate that will lead to large colonies appearing in a six‐week time frame. Furthermore, even in shallow Lake Taihu, Qin et al. (2018) measured a significant increase in average colony size over a short period of several days during a typhoon event with consistently high wind speeds, a result consistent with our findings.

This observation has profound consequences on the subsequent formation of surface blooms. Shortly after large wind events, the newly large colonies will be able to overcome turbulent mixing that the previously small colonies could not, leading to drastically different vertical transport results. Since blooms typically consist of large colonies (Cao & Yang, 2010; Wu et al., 2020; Zhu et al., 2014), this also means short periods of mixing via large wind events could act as a necessary precursor to surface harmful algal bloom formation as long as algal concentrations are high enough (see discussion below and Section 4.3 for more details). In a laboratory mesocosm experiment, Wu et al. (2019) found that increasing wind (up to 3.6 m/s) increased the volumetric median colony diameter at the water surface. Field experiments by Yang et al. (2020) found that intermittent wind‐induced disturbance favored (i) larger colony sizes, (ii) higher biomass, and (iii) stronger dominance of *Microcystis* over constant quiescent or constant wind conditions. We believe this result agrees nicely with our conclusion that wind is necessary to promote aggregation, quiescent conditions are necessary for algal growth, and the combination of the two in subsequent order is a recipe for a harmful algal bloom.

In regards to the sensitivity of aggregation to the initial algal concentration, the inversely proportional relationship between algal concentration and time to large colony appearance, *τ*_101_, has been documented in previous studies of marine snow. Jackson (1990) found their large‐sized colonies appeared within half a day of algal concentrations reaching 10^8^ cells/m^3^, a rate in line with the results described in this manuscript (Figure 10). We relate *τ*_101_ to initial concentrations only, but that is simply because we have a conserved number of total cells in our system. If instead we had growth and/or decay terms, we could track *τ*_101_ as a function of instantaneous algal concentration. By maintaining conservation of mass, however, we can clearly see that any location in the water column with algal concentrations on the order of 10^7^ cells/m^3^ will take over 10 days to form large colonies, whereas locations with concentrations on the order of 10^8^ cells/m^3^ will have large colonies within a day.

Since higher densities would lead to increased collisions, this finding is unsurprising from a physical standpoint; however, it does provide some important biological modeling insight. Regardless of wind conditions, aggregation will be negligible until algal concentration exceeds 10^7^ cells/m^3^. After this threshold is reached, the rate of aggregation will increase as concentration increases. A large wind event later in the season—when algal concentrations are high—will therefore have dramatically different aggregation consequences than a large wind event in the beginning of the season, when algal concentrations are low. Further, nonuniform algal concentration profiles will lead to nonuniform aggregation. Any depth where there is a peak in algal concentration will also act as a hot spot for aggregation, leading to nonuniform colony size distributions within the water column.

### 4.2 An evaluation of model assumptions

Before addressing the implications of these findings on harmful algal blooms, we must discuss how model assumptions may impact results. Let us start with our neglect of disaggregation and our limitation on maximum colony size. Large colonies (*d*_*k*_ > 420 μm) would almost surely fragment under our high wind/strong turbulence conditions (O'Brien et al., 2004). The fact that turbulence also promotes aggregation through enhanced mixing represents a colony size trade‐off. Turbulence causes the colony size distribution to skew towards the largest stable colony size, but the largest stable colony size decreases with increasing turbulence. If we were to allow larger colony sizes in the model, we would have to include fragmentation, a conclusion arrived at by Ackleh and Miller (2018) as well. Byrne et al. (2011) derived postfragmentation density functions for fractal bacterial flocs of *Klebsiella pneumoniae* in laminar flow, which indicated the number of postfragmentation flocs increases with increasing shear. A similar analysis could be conducted for *M. aeruginosa* and other colonial and motile harmful algae. Based on the results of Byrne et al. (2011), we would expect fragmentation to seed more small colonies in the surface mixed layer than in the metalimnion, which may balance out some of the heightened aggregation in the surface mixed layer.

Another constraint of this model is the restriction of algal growth, which is negligible over short timescales but significant over seasonal timescales. Recall this decision was made because *M. aeruginosa* colonies tend to be fractal in shape, and fractal aggregates are often the result of aggregation due to collisions instead of cell growth (Xiao et al., 2018). However, in experiments, Duan et al. (2018) found that *Microcystis* colony size significantly increased with increasing temperature. Although the aggregation kernel related to Brownian motion scales linearly with temperature (Equation (2)), this thermodynamic dependency alone cannot explain this variability. For the strains of *Microcystis* being investigated in the experiments, it seems increased algal growth with increasing temperature is responsible for the increase in colony size. In deriving our model, we have previously assumed aggregation due to cell growth is negligible, but this may not be true during peak surface water temperature conditions, leading to an underestimation of average colony diameter during high‐temperature conditions. To account for cell growth in future iterations of this model, the method of Ackleh and Miller (2018) for calculating cell growth within a colony–where only a certain proportion of cells along the edge of the colony are able to reproduce new cells–should be incorporated into Equation (7).

If we consider that quiescent conditions are hypothesized to be an immediate precursor to surface HABs (Michalak et al., 2013), then incorporating a growth term would likely change our results for intermittent high wind events (Figure 8c,d). We would expect slower frequencies of wind mixing to result in more opportunities for growth at the water surface during low wind conditions, leading to faster aggregation, which would cause a discrepancy between slow frequency and high‐frequency wind mixing not currently demonstrated in this model. Recall that Yang et al. (2020) determined that intermittent disturbance not only promoted aggregation in *M. aeruginosa* but total biomass as well.

### 4.3 Implications for harmful algal blooms

So far we have only discussed the mechanistic insight provided by the model into the vertical distributions of *M. aeruginosa*, but it is important to remember the ecological consequences of this insight. Surface HABs are mostly comprised of large colonies. Because wind‐induced mixing increases the rate of aggregation, we can think of large wind events as a necessary precursor to *Microcystis* bloom formation. Mainstream consensus on cyanobacteria HABs states that quiescent conditions are necessary for bloom formation (Michalak et al., 2013). While this may be true immediately preceding bloom formation, it is also true that there must be enough large wind events before the quiescent period to encourage aggregation in order for a surface bloom to form. But, the occurrence of large wind events is still not enough: These wind events must occur when algal concentrations exceed 10^8^ cells/m^3^ in order for large colonies to form within a day. In addition to modeling concerns, this finding has implications for water quality management. If water samples are taken from well above the photic depth in a lake dominated by motile and colonial cyanobacteria, algal concentrations will likely be low and the average colony size will likely be quite small, which may give the appearance that HAB formation is unlikely. Meanwhile, large colonies could be rapidly forming at subsurface algal concentration peaks near the photic depth, indicating a surface bloom is imminent.

### 4.4 Future work

A major objective of a mechanistic model is to generate hypotheses that drive further research. The results of this model suggest the need for a subsequent field study where meteorological conditions, lake thermal profiles, and both *Microcystis* concentration and colony size are tracked over depth and time at a relatively high frequency. Once model results can be validated with field data, there are many further avenues of the study suggested by the model, both from an ecological and numerical perspective. One major ecological concern of *M. aeruginosa* is the ability to produce and release microcystins, a cyanotoxin. Microcystins are known to increase in extracellular concentration when *Microcystis* is stressed, and they also seem to have a relationship with extracellular polysaccharide content and colony size (Hu & Rzymski, 2019; Li et al., 2020; Rzymski et al., 2020; You, 2020). In fact, it is even hypothesized that microcystins can trigger colony formation via quorum‐sensing processes (Rzymski et al., 2020). This raises an important question: How might the coupling of microcystin‐triggered quorum sensing with colony dynamics improve model predictions of both the spatial heterogeneity of *M. aeruginosa* biomass and extracellular microcystin concentrations? After all, *M. aeruginosa* is a threat to public health because they release microcystins. In this regard, the fundamental question is not necessarily where the *Microcystis* is, but where the microcystins are.

Keeping in mind that the goal is to improve predictions over a seasonal time scale, then it will be necessary to use our model as a subroutine—in addition to a subroutine for disaggregation—in larger modeling software that can handle hydrodynamics, biogeochemical cycling, and algal life cycles (e.g., AEM3D (Hodges & Dallimore, 2016) or Delft3D‐WAQ (Q. Chen & Mynett, 2006)). Since this model demonstrates aggregation is negligible except during high wind events at high algal concentrations, future models could also include a term that switches aggregation off when those conditions are not met. It would also be worthwhile to use these results to instead explore the evolution of the average colony size, ${\bar{d}}_{k}$, as a function of algal cell concentration and turbulence intensity. The model proposed in this manuscript is necessary to gain biological and physical insight into algal aggregation processes, but it may be possible to reduce some complexity once the system is understood. Aggregation processes mostly affect buoyant transport, which is governed by the colony diameter‐dependent settling velocity described in Equation (13). By restructuring the modeling in this way, the system of *k* equations can be avoided and bulk parameters remain the focus, removing most of the numerical expense that would be added by incorporating Equation (7) as a subroutine in software like AEM3D.

While the model described here has been derived for *M. aeruginosa* specifically due to their ubiquity and ecological importance, the modeling framework can easily be applied to any motile and colonial phytoplankton species. Different species have different motility and sticking mechanisms, so calculations of the advective velocity, *w*_*k*_(*z*,*t*), and sticking probability, *α*(*i*,*j*), will need to be tailored to each individual species. *M. aeruginosa* uses intracellular gas vesicles and buoyancy regulation mechanisms to achieve vertical motility, but many species of green algae use flagella to move about the water column, as an example. Despite these differences in subroutine calculations, the theoretical framework will remain largely unchanged from species to species and lake to lake. To promote the use of this model for different algal species, editable and annotated Matlab code used to simulate the base case scenario in Section 3.1 can be found at the Data Repository for the University of Minnesota (DRUM).

## 5 CONCLUSION

We have developed a theoretical model that tracks the meteorological‐driven movement and aggregation of *M. aeruginosa* in lake water columns. There are limitations in this model—in particular, disaggregation is not accounted for and no explicit validation with field data has been made. However, the process of constructing the model and the predictions generated by the model provide important insights into the possible drivers of harmful algal blooms. First, we have demonstrated that Smoluchowski aggregation qualitatively represents the colony dynamics of *M. aeruginosa*, and the coupling of transport and colony dynamics is an important mechanism of *M. aeruginosa* population models in stratified lakes. Further, the model is capable of generating the diurnal migrations exhibited by large colonies of *M. aeruginosa* to a depth of preferred light intensity, but small colonies are susceptible to turbulent entrainment and generally become well‐mixed throughout the surface mixed layer. Model results also clearly demonstrate that wind‐induced mixing and algal concentrations exceeding 10^7^ cells/m^3^ are necessary to promote the aggregation of an initial single cell population to an algal population dominated by large colonies (*d*_101_ = 160 μm) within 6 weeks, a time scale in accordance with field measurements (Cao & Yang, 2010; Xiao et al., 2018). This finding suggests quiescent conditions alone are not sufficient for surface bloom formation of colonial and motile harmful algae—large wind events prior to quiescent conditions are an important necessary precursor. In addition, the model provides guidance for future field data collection and model studies (e.g., quantifying the roles of extracellular polysaccharide and microcystin content as they relate to aggregate sticking probability). To practically implement the results of this theoretical model, we have identified ways to (i) incorporate this model into larger software in computationally efficient ways, and (ii) extrapolate this theoretical framework to different algal species.

## AUTHOR CONTRIBUTIONS

**Jackie Taylor:** Conceptualization (lead); data curation (equal); formal analysis (lead); funding acquisition (equal); investigation (lead); methodology (lead); software (lead); validation (lead); visualization (lead); writing – original draft (lead); writing – review and editing (equal). **M. Carme Calderer:** Conceptualization (supporting); methodology (supporting); writing – review and editing (equal). **Miki Hondzo:** Conceptualization (supporting); data curation (equal); formal analysis (supporting); funding acquisition (equal); investigation (supporting); methodology (supporting); supervision (equal); writing – review and editing (equal). **Vaughan R. Voller:** Conceptualization (supporting); formal analysis (supporting); funding acquisition (equal); investigation (supporting); methodology (supporting); supervision (equal); writing – review and editing (equal).

## CONFLICT OF INTEREST

The authors cannot identify any potential conflicts of interest.

### OPEN RESEARCH BADGES

This article has earned Open Data and Open Materials badges. The data will be uploaded to the Data Repository for the University of Minnesota upon article acceptance. In the meantime, data has been included in the submission packet. Matlab code to run the model will be uploaded to the Data Repository for the University of Minnesota upon article acceptance. In the meantime, the code has been included in the submission packet.

## Data Availability

Data archiving is underway at the Data Repository for the University of Minnesota (DRUM), where interested parties can find lake thermal and hydrodynamic profiles from Ramsey Lake, MN, and an example Matlab simulation script.

## APPENDIX A Calculation of the buoyant velocity

Following the work of Medrano et al. (2013), we can calculate the change in individual cell density, *ρ*_cell_, in light conditions by

(A1)
$$\frac{d\rho_{\text{cell}}}{dt}=\frac{a}{60}{\mathrm{Ie}}^{-I/I_{0}}+d$$

and in dark conditions by

(A2)
$$\frac{d\rho_{\text{cell}}}{dt}=-b\left(\rho_{\text{cell}}-\rho_{\mathrm{ref}}\right)H\left(\rho_{\text{cell}}-\rho_{\mathrm{ref}}\right)$$

where *I*(*z*,*t*) is the irradiance, *I*_0_ is the irradiance such that *ρ*_cell_(*I*_0_) is maximum, *d* is the background rate of density change when *I* = 0, *ρ*_ref_ is the minimum cell density below which cells will not reduce their carbohydrate content, *a* is a normative factor, *b* is the slope of density change in the dark, and *H*(*x*) is the Heaviside function defined to be unity when the argument *x* is positive and zero otherwise. We used a value of *I*_crit_ = 5.75 W/m^2^ to differentiate between light and dark conditions and an initial single cell density of *ρ*_0_ = 1060 kg/m^3^. For all parameter values, see Table A1.

**TABLE A1:** Parameters used to calculate the buoyant velocity

Variable	Description	Value
*a*	Normative factor in Equation (A1)	4.96 × 10^−5^ s^2^/m^3^
*I*_0_	Irradiance such that *ρ*_cell_(*I*_0_) achieves a maximum	146.43 W/m^2^
*d*	Background rate of density change when *I* = 0	−2.75 × 10^−4^ kg/m^3^/s
*b*	Slope of density change in dark conditions given by Equation (A2)	1.58 × 10^−5^ 1/s
*ρ*_ref_	Critical density where cells will no longer reduce their carbohydrate content	1037 kg/m^3^
*I*_crit_	Critical light intensity differentiating between light and dark conditions	5.75 W/m^2^
*ρ*_0_	Initial density of single cells	1060 kg/m^3^
*n*_gas_	Ratio of gas vesicle volume to colony volume	7%
*n*_cell_	Ratio of cell volume to colony volume	*n*_cell_(*k*) = −0.0073 *k* + 0.9373 (%)

We assume that the cell density of each individual cell, whether it belongs to a colony or not, will react to the instantaneous light intensity in every grid cell at every time step by these equations. To calculate colony density, *ρ*_*k*_, from single cell density, we use the relationship

(A3)
$$\rho_{k}=\rho_{\text{cell}}n_{\text{cell}}\left(k\right)\left(1-n_{\mathrm{gas}}\right)+\rho_{\mathrm{muc}}\left(1-n_{\text{cell}}\left(k\right)\right)$$

where *n*_cell_(*k*) is the ratio of cell volume to colony volume, *n*_gas_ is the ratio of gas vesicle volume to colony volume, and *ρ*_muc_ is the density of cell mucilage (Medrano et al., 2013). Like Medrano et al. (2013), we have kept *n*_gas_ and *ρ*_muc_ constant for all simulations; unlike Medrano et al. (2013), *n*_cell_ is not constant but will vary with colony size because we assume all *M. aeruginosa* colonies have a fractal geometry and the relative content of mucilage to cells will increase with increasing cell size (Zhu et al., 2014). For parameter values, please see Table A1. Once *ρ*_*k*_ has been calculated for all values of *k* at every time step in every grid cell, we can then calculate the buoyant velocity, *w*_*k*_(*z*,*t*), by Equation (13).
